# 2013 *JRR* Terashima Award

**DOI:** 10.1093/jrr/rrt126

**Published:** 2013-11

**Figure:**
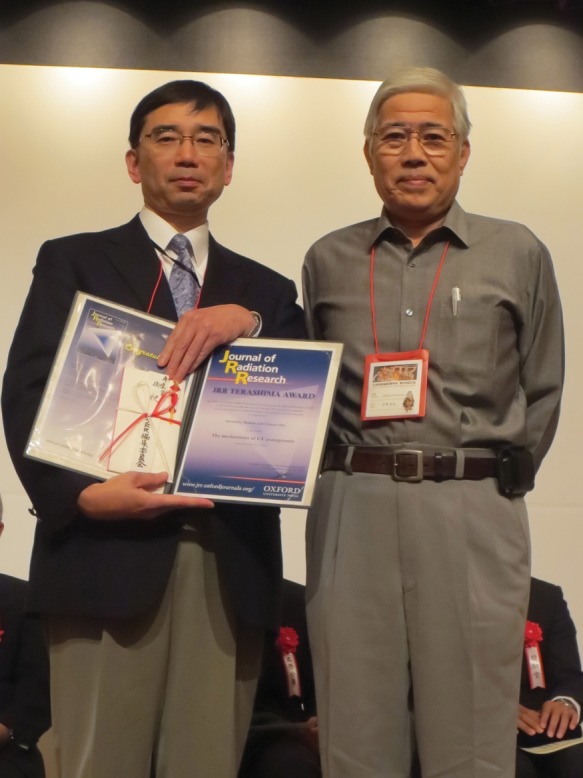

The *Journal of Radiation Research (JRR)* is pleased to announce this year's winner of the 2013 *JRR* Terashima Award:

Title: **The mechanisms of UV mutagenesis**

Authors: **Hironobu IKEHATA, and Tetsuya ONO**

Graduate School of Medicine, Tohoku University

*J. Radiat. Res*., 52(2), 115–125 (2011)

## Comment from the Editor-in-Chief

Congratulations on winning! The *JRR* Terashima Award was established in 2002 to every year honor the article contributing the most to the journal that year, and it is funded by Dr. Toyozo Terashima's contribution to The Japan Radiation Research Society (JRRS). The winner was limited to a basic research scientist who belonged to the JRRS for the first 10 years. This is the first year where JASTRO members have had the opportunity of winning the Terashima Award since JASTRO partnered with *JRR* in 2009. The winners were awarded a certificate and 100,000 yen, and will have the article processing charge waived for their next paper in *JRR*.

## Comment from the Author

It is an unexpected pleasure and a great honor for us to receive the Terashima Award for our latest review paper published in *JRR*, in which we reviewed the updated overview of ultraviolet radiation (UV)-induced mutagenesis from the molecular mechanisms, the mutation spectra to the manifestations in cells and skin. We provided in the review a few specific figures of molecular mechanisms illustrating each UV mutagenesis pathway as well as a summary figure of the overview of UV mutagenesis, all of which we believe helps readers to easily understand our points. This review also characterized UV mutations in terms of the effect of UV wavelengths, which has been one of the major subjects of our research for years. Comprehensive reviews on UV mutagenesis have been lackingin the literature of mutation research, which made us write this review. We are unexpectedly pleased to find it accepted by a large audience in our research fields, which is exemplified by its remarkably frequent citations.

